# Future of Chondroprotectors in the Treatment of Degenerative Processes of Connective Tissue

**DOI:** 10.3390/ph13090220

**Published:** 2020-08-28

**Authors:** Stanislav Sukhikh, Olga Babich, Alexander Prosekov, Nikolai Patyukov, Svetlana Ivanova

**Affiliations:** 1 Institute of Living Systems, Immanuel Kant Baltic Federal University, A. Nevskogo Street 14, 236016 Kaliningrad, Russia; stas-asp@mail.ru (S.S.); olich.43@mail.ru (O.B.); sved_08_89@mail.ru (N.P.); 2 Department of Bionanotechnology, Kemerovo State University, Krasnaya Street 6, 650043 Kemerovo, Russia; 3 Laboratory of Biocatalysis, Kemerovo State University, Krasnaya Street 6, 650043 Kemerovo, Russia; a.prosekov@inbox.ru; 4 Natural Nutraceutical Biotesting Laboratory, Kemerovo State University, Krasnaya Street 6, 650043 Kemerovo, Russia; 5 Department of General Mathematics and Informatics, Kemerovo State University, Krasnaya Street 6, 650043 Kemerovo, Russia

**Keywords:** osteoarthritis, chondroprotectors, treatment, collagen, chondrocytes, hyaluronic acid

## Abstract

Osteoarthritis is one of the most common diseases of the connective tissue of the elderly. It was found that most epidemiological studies used the Kellgren and Lawrence system for classification of osteoarthritis, which indicates one of the 5 degrees (0–4) of osteoarthritis in various joints according to the radiographic atlas. It has been proven that chondroprotectors are represented by the following active substances: chondroitin sulfate, glucosamine sulfate or hydrochloride, hyaluronic acid, glycosaminoglycans, extraction preparations from animal or plant raw materials. The sources of raw materials for the manufacture of combined chondroprotectors are known, methods for their preparation and use are described. The main drugs on the chondroprotective market are presented. The effectiveness of their use for the treatment of osteoarthritis has been proven. It was found that preparations containing chondroitin sulfate have anti-inflammatory activity, affecting mainly the cellular component of inflammation, stimulate the synthesis of hyaluronic acid and proteoglycans. Methods of treating osteoarthritis using cell therapy (the use of readily available, highly proliferative, and multipotent mesenchymal stromal cells) are presented.

## 1. Introduction

The population aging process is steadily accelerating not only in Russia but also in most countries of Europe. Already now in many regions, people over 60 make up more than 30% of the population. The proportion of older people in the general structure of the population is growing faster than in any other age group.

One of the most common diseases of the connective tissue of older people is osteoarthritis (OA). Osteoarthritis (OA) (synonyms: osteoarthrosis, erosive (osteo)arthrosis) is a degenerative-dystrophic disease of the joints, which is caused by the damage to the cartilage tissue of the articular surfaces. The symptom that primarily characterizes the disease is pain, which may be accompanied by difficulty in movement and deformities. The most affected joints are the hips, knees, and shoulders. OA is the most common joint disease characterized by progressive cartilage degeneration, changes in the subchondral bone, and chronic synovitis [1,2,3]. Modern treatment of OA is limited to the use of drugs that affect the symptoms of the disease: analgesics, anti-inflammatory drugs, and devices for treatment [4,5,6,7].

It is believed that bone development, growth, and regeneration occur in the phases of endochondral ossification. First, cartilage cells and the surrounding matrix form a frame for bone formation [8]. Then osteoblasts penetrate this matrix and precipitate the mineralized parts of the bone. Despite the long-term popularity of this model, it does not describe all the circumstances of the regeneration of the mesenchymal skeleton. A number of studies have established that primitive chondrocytes not only build up a temporary matrix, but can also differentiate into cartilage, mature bones, and even into related stromal reticular cells [9,10]. As the bone lengthens, hematopoietic stem cells move from the liver to the bone marrow, supporting mature mesenchymal stem cells of the created population of the perisinusoidal niche [8]. The articular cartilage, clamped between the synovial fluid and the advancing subchondral bone, becomes more and more distant from the bone marrow itself with increasing ossification. This creates an articular compartment in a unique setting for a unique function. Articular cartilage deserves special attention when searching for new cellular therapies for OA treatment.

OA can be defined as degenerative processes in the connective tissue. Degenerative-dystrophic changes in the connective tissue are diseases of the musculoskeletal system and connective tissue (OA, osteochondropathy, dorsopathy, degenerative-dystrophic changes in the bone-cartilage structures, consequences of injuries, dysplastic diseases; soft tissue diseases (ligaments, tendons, muscles)), various deformations of the musculoskeletal system, etc.

Key symptoms are determined by joint pain and stiffness. Joint pathology is varied and includes focal damage, synovial swelling and inflammation, osteophytes (bone spurs), weakening of the periarticular muscles, ligamentous weakness, abnormal remodeling, and thinning of the subarticular bone, and loss of articular cartilage. It is widely believed that OA is an age-related dynamic response of a joint to injury or inflammation. All joint tissues are damaged by OA, but the most noticeable are the articular cartilage loss and changes in the adjacent bone. OA is the destruction of the joint as an organ, which in terms of its effects on the body can be compared to renal or heart failure.

The purpose of this review is to classify OA by severity and to study the effectiveness of known drugs with chondroprotective properties on the course of OA.

## 2. Results and Discussion

Inaccuracies in the description of the OA radiographic have led to studies using conflicting criteria. Besides, studies pay too much attention to the presence of osteophytes.

In recent studies, the general radiographic classification was systematized according to radiographic characteristics, the criteria were described and the reproducibility and clinical correlates of each of them were evaluated. Good observational reproducibility was established for the articular space narrowing of the knee joint, hip joint and hand, osteophytosis, and the overall Kellgren-Lawrence grades; the diagnosis of osteophytosis [11,12,13] and OA of the hip [14] is closely related to the presence of pain in the knee or hip joint, respectively. Criteria for the OA severity are presented in Table 1 and are associated with the consecutive occurrence of osteophytes, narrowing of space, sclerosis, and cysts.

This classification system has two important limitations [10]. Modern atlases of standard radiographs provide a more consistent approach to the classification of these individual features, ensuring greater extrapolation between results from different studies [15,16].

The main OA diagnostic technique is based on the criteria developed by the American College of Rheumatology (ACR) [2,3]. Diagnostic criteria identify patients with clinical OA by joint pain on most days of the previous month (primary criterion).

In 1952, Kellgren and Moore described a generalized OA condition in which a polyarticular disease is connected to Heberden nodes [15,17,18]. The affection of large joints, e.g., hip joint, is accompanied by diffuse (bilateral and concentric) loss of cartilage. The symmetry of joint damage in this disorder is pronounced. The classification of a subset of erosive OA is considered in some patients with the development of interphalangeal joint erosion [19]. This is an acute disorder with signs of inflammation, subsides over months or years, is accompanied by joint deformity and ankylosis, usually occurs in middle-aged women. Recent studies have found evidence that erosion in OA may simply be an aggressive form of destruction of joints that already require assistance [18,19].

Currently, many scientists have begun to consider OA as a metabolic and as a calcium-deficient disease [1,20,21,22,23]. In OA, there are violations of the synthetic activity of chondrocytes (ultrastructural changes); decreased hydrophilicity of cartilage; slow degeneration, and a decrease in the number of chondrocytes occur, which leads to the loss of normal biomechanical properties by the cartilage matrix [4]. An increase in the content of lysosomal enzymes released as a result of the death of chondrocytes destroys the structure of the cartilage. Released proteoglycans, as well as collagen and chondrocyte breakdown products, are strong antigens. A secondary inflammatory process of an autoimmune nature develops as a closed pathological circle [4,11]. Damage to the vascular network that feeds the subchondral bone leads to intraosseous hypertension with focal hypoxia and bone ischemia. Due to the destruction of the cartilage, subchondral bone tissue is subjected to significant pressure, in which trabecular microcracks and micro-fractures, sclerosis, and osteophyte formation occur [14,24].

Both plant and animal products have chondroprotective characteristics.

Turmeric (or curcumin) is an extract of turmeric, a yellow spice, and a member of the Zingiberaceae family. Both turmeric and ginger have roots in Ayurvedic and Chinese medicine [25], and turmeric exhibits anti-inflammatory effects through cyclooxygenase-2, inhibition of prostaglandins, and leukotoxins. There is a wide variation in daily doses from 180 to 2000 mg, which makes direct comparison problematic.

A meta-analysis published in 2016 included the results of 4 placebo-controlled studies of knee OA; 2 studies against ibuprofen and 1 against diclofenac in the therapy of this disease. Regardless of several idiosyncratic side effects reported in these studies, the meta-analysis established safe daily dosage of turmeric (4800 mg/day during 4 months) [26].

Harpagophytum (devil’s claw) is an African plant possessing anti-inflammatory effects (similar to turmeric) due to inhibition of leukotoxin. The study results (Gagnier et al.) of the role of Harpagophytum in the treatment of lower back pain and OA in three randomized placebo-controlled trials with 385 volunteers with OA of hip and knee joints was described in [26]. The effectiveness of harpagophytum powder at a dose of 60 mg was established, although the need for longer and more varied tests to be able to recommend it for clinical practice was mentioned.

Many plant and animal products, such as green tea and fish oil [27], have traditionally been used by patients with OA to relieve pain, which by definition makes them nutraceutical. There is new evidence that the effectiveness of these natural products for pain relief in OA depends on certain active ingredients that are enriched with these natural sources, such as epigallocatechin-3-gallate, enriched in green tea [28].

For example, nutraceutical products made from green tea include green tea powder, green tea extract, and concentrated epigallocatechin-3-gallate (EGCG) derived from green tea. Since ancient times, turmeric and ginger have been used in India and China to treat OA, in particular, to treat pain in OA [22,23]. New Zealand has a centuries-old practice of using green lip mussels for the same purpose [29]. Despite the lack of a complete understanding of the action mechanism of nutraceuticals on pain in OA, there is more and more evidence of their pronounced anti-inflammatory effect [30].

Preclinical studies confirmed chondroprotective, anabolic, and anti-catabolic properties of avocado-soybean unsaponifiables (ASU), while clinical trials showed their effectiveness in pain alleviation in humans. The active component has not yet been established; it is generally accepted that sterol is responsible for most of the biological activity of ASU in the articular chondrocytes [31]. The results of a 3-month clinical study describe that daily intake of different doses of ASU significantly reduced both NSAID intake and Lesquesne pain score [32]. A study [33] demonstrated the effectiveness of a daily intake of 300 mg piaskledin (100 mg of avocado and 200 mg of soybean oil) for six months, which is similar to intake of chondroitin sulfate against pain, the decrease in score for the Western Ontario and McMaster Universities Arthritis Index (WOMAC) corresponded to 50%. Repeated examination of patients 2 months after the trial did not reveal a prolonging effect of therapy. As a result of a large-scale observational study in Poland, patients taking ASU daily for 6 months showed a significant improvement in Lesquesne and VAS scores [34]. It is hypothesized that ASU treatment not only affects pain but also has a structural-modifying effect in OA of the hip joint. 3 years after the staged ASU treatment there was 20% less joint loss among participants compared to the placebo group [35].

In a cross-sectional clinical study, patients took 333 mg *Boswellia serrata* extract (BSE) capsules three times a day for 8 weeks. The results showed a significant reduction of edema (in the absence of radiographic changes), knee pain, improved knee flexion, and an increase in walking time. The composition of the extract was determined by HPLC [36] (boswellic acid 40%, the main potential active ingredient (3-*O*-acetyl-11-keto-beta-boswellic acid) only 2%). In another study, patients were administered the drug (5-loxin, enriched with *Boswellia serrata* extract with 30% AKBA) in two doses: 100 mg/day and 250 mg/day. In each case, there were changes in pain, physical function, as well as WOMAC and Lesquesne scores. An improvement in WOMAC scores in the high-dose group was observed as early as 7 days [37]. A burning sensation was reported (67% of patients), but this did not cause discontinuation of therapy [38]. Zucapsaicin, a synthetic capsaicin *cis*-isomer, acts similar to capsaicin, but with higher tolerance [39]. Participants in a 12-week study [19] used zucapsaicin cream at concentrations of 0.075% and 0.001%. The positive effect in terms of pain and WOMAC scores was achieved, but when the cream was used at a higher concentration, the effect was more pronounced. It was found that the effect of zucapsaicin persisted in repeated studies after 52 weeks.

Ginger has been widespread in medical practice (nausea, arthritis, and vascular disease) for thousands of years, especially in China. The pungent taste of the plant is due to the content of volatile oils, especially phenol-gingerol [39]. The ginger extract tablets were administered to patients for 6 weeks. There was a decrease in pain in standing position, pain after walking, and WOMAC scores [38]. It was found that the consumption of ginger powder even in a small dosage (1 g/day) reduces inflammation markers in patients. Serum nitric oxide (NO) and C-reactive protein concentrations decreased in patients after 3 months of supplementation.

Polyphenols have positive health effects and are found abundantly in plant sources. The anti-inflammatory and antioxidant activities of tea and pomegranate polyphenols in vitro are known, and recent studies in humans have shown efficacy in relieving pain in OA patients [16,38]. Green tea has been widespread in China and Japan for thousands of years [40]. It is known for its antioxidant activity, which is 25–100 times higher than the antioxidant characteristics of vitamins C and E [41]. Most of the activity in green tea is determined by catechins, especially epigallocatechin-3-gallate (EGCG). It was clinically determined that a daily intake of 200 mL of green tea without sugar for two weeks improved WOMAC scores, physical function, and stiffness in patients with OA [31]. It is believed that pomegranate juice polyphenols, tannins, and anthocyanidins, which are considered as therapeutically active ingredients, have antioxidant and anti-inflammatory effects [35]. Phenolic compounds such as oligomeric proanthocyanidins or oligomeric procyanidins (OPC), found in pine bark (patented as pycnogenol), in grape seed extract, in cinnamon, as well as in the peel, seeds or bark of other plant components, have therapeutic activity. There is evidence of pain relief and general improvement in OA patients who were taking 100 mg of pycnogenol for 3 months in several clinical trials.

It is known that in terrestrial and marine animals about one-third of the total amount of nitrogenous substances is accounted for by collagen compounds. The term “collagen” comes from the Greek words “colla”—glue and “genau”—producing. This concept is associated with a widespread group of proteins found only in animals. Connective tissue fibers are formed from collagen. Collagen is always concentrated in the connective tissues of marine and terrestrial animals—in the skin, bones, fins, tendons, and other parts and organs of animal organisms. The most important types of connective tissue are known: elastic tissue (tendons), tightly bound tissue (skin), cartilage (trachea), bone tissue (dentin, bones), chord supporting tissues (intestines), reticular connective tissue (nerves, capillaries) [42].

Collagen-containing raw materials are mainly connective tissue, consisting of cells, intercellular substance and collagen fibers, in addition, it contains a small amount of elastin and reticulin fibers, as well as passing blood vessels. Connective tissue provides strength to the external and internal structures of the animal organism, it is rich in valuable mineral substances, contains amino acids, physiologically active substances in sufficient quantities [25].

According to their purpose, collagen-containing raw materials are divided into those intended for the production of gelatin and glue, for the production of Belkozin sausage casings, for the production of special-condition glue, for medicine, for the production of various protein products—flour, feed, fertilizers, hydrolysates [41].

Collagen, depending on the type of source of origin, is divided into fibrous collagen of the dermis of skins and tendons, hyaline collagen of bone tissue—ossein, chondrin collagen of cartilage, ichthyulin collagen of a fish bladder—ichthyocol, and fish fin collagen—ichthylepiline [28].

The amino acid composition of collagen is characterized by the obligatory presence of oxyproline, which is like a sign of connective tissue, and the absence of tryptophan, which is a sign of any muscle tissue. By the amount of oxyproline, you can calculate the collagen content in anybody [8]. This is used in biochemistry for recognition of collagen in various tissues and organs, in the food industry—for the detection of unacceptable waste in meat, in the gelatin industry—to control the purity of gelatin, in leather and fur production—to identify waste [29].

The collagen content in the organs and tissues of any living organism is different: the largest amount of collagen is concentrated in the bones, skins, cartilage, tendons, and intestines [39]. In the skin of pigs and dogs, the collagen content is 64%, in human skin—72%, in the skin of cattle—80%, in the skin of marine mammals—over 80%; a significant amount of collagen is found in the skin of fish; connective tissue of fat of whales contains from 74 to 88% of collagen, up to 83% in the fins of whales, and up to 89% in the tissues of spermaceti organ of a sperm whale [42]. The percentage of fish collagen-containing raw material is as follows: skin—2.0–12.6% (of the total mass of fish); fins—0.8–8.0%, swimming bladders—0.4–11.4%, scales—0.8–6.0%, bones—9–19%, sturgeon viziga (spinal chord)—7.6–10.2% [28].

The mass composition of the fins of whales (saivals and finials) has 1.7%, the bones—30.2%, the sperm whale fins have a mass of about 2%, and the mass of collagen-containing raw material of spermaceti organ of the sperm whale is about 8% of the carcass weight, which is about 2.5 t on average [43].

The most rational way to preserve collagen-containing raw materials is freezing.

A very wide range of food, medical, fodder, and technical products are obtained from collagen-containing raw materials: glue, gelatin, shells, films, sponges, suture material, prostheses, skin, furs, various fodder products [7]. Fish glue from swim bladders was widely used at the dawn of aircraft manufacturing, and special glue from fish skin was used in the production of picture tubes for color TVs. Porous collagen films and sponges from collagen-containing raw materials of whales were successfully tested in the burn center of the A.V. Vishnevsky Institute of Surgery, shoes were made from the skin of whales, the skins of sharks and other fish were used to make various leather products, the skins of sea lions and other marine mammals were used to obtaining valuable fur [43].

The resources of industrial collagen raw materials of both terrestrial animals and aquatic organisms are very large. However, the collagen-containing resources of terrestrial animals have been studied more thoroughly than the raw materials of aquatic organisms [17].

In a study [30], no statistically significant intergroup differences in the effectiveness of treatment with undenatured collagen, an alternative form of collagen, glucosamine, and chondroitin were found in groups of 26 patients.

Martin et al. [29] presented the results of an analysis of the effectiveness of oral collagen intake in various doses (8 trials of 3 doses of collagen hydrosilicates, gelatin versus placebo, and undenatured collagen versus glucosamine hydrochloride and chondroitin sulfate). A low level of research methodology for OA therapy was revealed. Despite the widespread distribution of collagen drugs (oral, intra-articular form) in the therapeutic practice of many countries, the evidence base for the effectiveness of these drugs has not yet been collected due to the weak correlation between drug intake and the main symptoms of the disease (pain sensations, dynamics of motor function).

High moisture-binding capacity and structure-forming properties determine the main functions of glycosaminoglycans (GAG). In general, the amount of GAG correlates well with the resistance of the articular cartilage to deformation [4]. There are four main GAG classes in cartilage (hyaluronan, keratan sulfate, dematan sulfate, and chondroitin sulfate). More than 80% of GAG in articular cartilage is chondroitin sulfate (CS), which has gel-like characteristics (lubrication, water retention, load resistance) [5,6,7]. The two most common disaccharide isomers among CS are chondroitin-4-sulfate and chondroitin-6-sulfate.

To date, only a few studies of CS have been conducted in patients with OA [20]. Sulfotransferases involved in the sulfation of cholesterol are divided into three types.

Some of these enzymes cause pathological genetic diseases, for example, Ehlers–Danlos syndrome [10] and Kashin–Beck disease [21]. These clinical and laboratory observations have shown that the deregulation of the sulfate group in CS can cause abnormal CS structures and cartilage disturbance. As mentioned above, most existing studies of CS and OA are limited to small groups of patients and did not consider possible variations in cartilage microanatomy [9].

Chondroprotectors are represented by a small number of active substances:

- Chondroitin sulfate
- Glucosamine sulfate or hydrochloride
- Hyaluronic acid
- Glycosaminoglycans
- Extraction preparations from animal or plant raw materials.

The prescription of chondroprotectors is indicated at any stage of degenerative joint disease. CS-based bone and cartilage metabolism correctors have chondroprotective and chondrostimulating effects. CS is a sulfated GAG consisting of repeating disaccharide units of d-glucuronic acid and *N*-acetyl-d-galactosamine [5].

OA is associated with a local deficiency of certain substances, including CS, so its use in OA treatment is justified. CS has an anti-inflammatory effect, stimulates the synthesis of proteoglycans, collagen, and hyaluronic acid, reduces the catabolic activity of chondrocytes, and affects the metabolism of the subchondral bone [44].

A variety of drugs with chondroprotective activity features many different forms of release (for external and internal use, for injection, for intra-articular administration) and manufacturers of this group of drugs [45].

Systematization by the forms of release showed that most often chondroprotective drugs are available in the form of oral drugs (capsules, tablets, powders)—73.3%, injection forms (including for intraarticular administration)—13.3%, and soft dosage forms for external use (ointments, creams)—13.4% [44].

A variety of chondroprotectors in the Russian pharmaceutical market is also associated with the features of their registration. In this regard, there are prescription drugs (dosage forms for injections and intraarticular use) and over-the-counter drugs (oral preparations—tablets, capsules, powders), medical forms (most hyaluronic acid preparations for intraarticular use), dietary supplements (mainly combined preparations of CS and glucosamine sulfate or hydrochloride). Non-prescription chondroprotectors account for 61.0%.

It was revealed that the leaders in terms of sales among chondroprotectors in the world are Teraflex (Bayer Healthcare, Leverkusen, Germany), Alflutop (C.S. Biotehnos SA, Otopeni, Romania), Arthra (Unipharm, New York, NY, USA), Dona (Biologici Italia Laboratories S.R.L./Rottafarm S.p.A, Masate, Italy), Artro-Aktiv (Diod, Moscow, Russia), Flexinovo (Adamed Pharma, SA, Pieńków, Poland), Piaskledine (Laboratoires Expanscience, Paris La Defense, France), Structum (Laboratoire Pierre Fabre Médicament, Paris, France), Chondrogard (Soteks, Moscow, Russia), and others.Along with non-steroidal anti-inflammatory drugs (NSAIDs), chondroprotectors are the basis of a therapeutic approach to the treatment of patients with OA. The basis of the action of these drugs is the influence on the composition of the synovial fluid. What happens is that with the development of OA, clusters of pathological chondrocytes contribute to the production of a defective main substance of cartilage, which is partially depolymerized, with a low content of proteoglycans [46].

Chondroprotectors are mainly registered as biologically active additives or are OTC drugs, and therefore, when forming the range of drugs in this series, consumer preferences are playing a significant role [18].

The clinical efficacy of complex metabolic therapy, including the simultaneous use of a chondroprotector, an osteoprotector, and an angioprotector containing glucosamine sulfate and chondroitin sulfate, calcium hydroxyapatite, polyunsaturated omega-3 fatty acids, vitamins, minerals, extracts from medicinal plants was studied in 564 cases of patients with OA of the knee joint. As a result of treatment, the pain syndrome decreased significantly, motor activity in the main group of patients improved. For the long-term therapeutic effect, the prevention of exacerbations and the progression of gonarthrosis, regular use of complex metabolic therapy with courses from 3 to 6 months is necessary, and if necessary, continuous use is possible and safe [5].

Chondroitin sulfate is one of the most important main components of connective tissue, is part of the bone, cartilage, tendons, ligaments, and in many respects provides a mechanical function of the joint, in particular resistance to compression [1,2,20,43].

Scientists [3] studied the effectiveness of a drug that contains 500 mg of glucosamine hydrochloride and 400 mg of CS. The main group included 25 women aged 58.3 ± 5.6 years with OA of the knee joints of the II–III degree according to the Kellgren grading. All patients complained of pain in the knee joints, in 18 of them (72%) there was a limitation of mobility in the joints. The control group consisted of 17 patients with a similar diagnosis at the age of 56.8 ± 6.2 years who received treatment according to the traditional scheme, including NSAIDs during the first 2 weeks, an individual complex of physiotherapeutic exercises, physiotherapeutic measures, etc. In the main group, the traditional treatment regimen was supplemented with a drug with chondroitin sulfate. The drug was prescribed during the first month, one capsule three times a day, then 1 capsule twice a day. The total duration of treatment was 2 months. Evaluation of effectiveness was carried out using a McGill Pain Questionnaire, VAS, the severity of joint inflammation according to the Ritchie index. Patients taking chondroitin sulfate showed a significant decrease in the severity of pain, in particular, on a visual analog scale, the severity of pain in the main group decreased from 7.35 to 2.34 (*p* < 0.05), while in the control group the decrease was less pronounced (from 7.45 to 4.56). Side effects after taking the drug were not found [3].

Chondroprotectors are intended to solve the important problem of the disease: to prevent incapacitation and reduce disability. According to scientists [19], this led to the use of various drugs based on chondroitin sulfate and glucosamine sulfate for oral and parenteral methods of their use. The creation of a transdermal glucosamine complex allowed the drug to be injected precisely into the joint area. To achieve an anti-inflammatory effect and enhance penetration through the skin, ascorbic acid and dimethyl sulfoxide (DMSO) were introduced into the composition of the drug.

Most modern methods of therapy for musculoskeletal system diseases do not affect the cause of the disease, they affect the symptoms, eliminate pain. The possibility of influencing the etiological mechanisms of the musculoskeletal system diseases according to the principle of replacement therapy appeared with the development of chondroprotectors containing glucosamine and chondroitin sulfate (CS), belonging to the pharmacological group of correctors of bone and cartilage tissue metabolism [19]. An important role in maintaining the required osmotic pressure (elasticity of the matrix and collagen fibers) is played by CS, which is part of the articular cartilage and has a chondroprotective, chondrostimulating pharmacological effect and stimulates regeneration. Drugs containing CS act primarily on the cellular component of inflammation and stimulate the synthesis of hyaluronic acid (HA) and proteoglycans.

HA is a polysaccharide from the glycosaminoglycan family consisting of repeating disaccharide units of *N*-acetyl-d-glucosamine and glucuronic acid [22].

In the human body, HA is one of the main components of the intercellular substance, acts as a natural metabolite of connective tissue, and inhibits the action of proteolytic enzymes, blocking inflammation.

Among the drugs with chondroprotective action, created based on modified HA (MHA), proteinogenic and antioxidant amino acids. Release form: in syringes of 2.0 mL concentration of MHA makes 1.5%; in 5.0 mL vials, the concentration of MHA was 0.8% [1,2,20,43].

Contraindications to the intraarticular use of this drug: acute OA; synovitis; for extra-articular use: acute radiculopathy [21]. There are general contraindications for all forms of administration, which include hypersensitivity to the components of the drug (HA, ascorbyl phosphate); autoimmune diseases of the connective tissue; inflammation of the tissues in the injection area; coagulopathy and/or taking anticoagulant drugs; the presence of osteophytes in the joints [19].

In the vast majority of cases, the injections were well tolerated by patients and were not accompanied by any significant side effects [22].

Among the side effects noted: moderate local pain from a needle stick for several days after the procedure; increased pain in the patient immediately after the injection and its preservation after that; a slight aggravation of the process after the initial improvement (usually by the 2nd or 3rd day) followed by a good regression by the 7th day. According to the authors of the study, the varying severity of these manifestations is a heterogeneous picture of one pathogenetic state in people in the acute period of the disease with severe pain—the presence of acute inflammatory changes in the tissues [14,23,24]. It is known that HA metabolites under conditions of a pronounced inflammatory process possess not anti- but pro-inflammatory activity and can cause exacerbation [33]. The studies of leading scientists [25] for the treatment of OA present the experience of using biologically active additives obtained by enzymatic hydrolysis of the cartilaginous tissue of aquatic organisms. Biopreparations from various types of aquatic organisms are standardized for hexosamine content (at least 2%), contain chondroitin sulfate (6%), and collagen (16–24%) and are a complex of proteoglycans with high, medium, and low molecular weight. The hydrolyzate of cartilage tissue of aquatic organisms is toxicologically safe. In experiments and clinical practice, its anti-inflammatory and chondroprotective effects were established. The use of hydrobionts of hydrolyzed cartilage tissue is most appropriate in cases where NSAIDs are not effective enough [26].

The success of using hydrobionts of cartilage hydrolyzate [30] has also been confirmed by a reduced need for NSAIDs. So, eight weeks after the course of therapy, 67% of patients completely refused to take NSAIDs due to the absence or significant reduction of pain and stiffness, 33% of patients continued to take NSAIDs only on demand (up to 2 times a week), of which 10% of patients reduced daily dose: nimesulide—up to 100 mg; meloxicam—up to 7.5 mg.

Most patients appreciated the therapeutic effect of hydrobionts of cartilage hydrolyzate. Improvement was observed in relation to certain symptoms of OA. So, the intensity of pain during walking and at the beginning of the movement significantly decreased, and the number of episodes of pain at rest and night also decreased. Patients were less likely to experience waking at night due to pain and joint pain when waking in the morning. The frequency of neuropathic pain complaints has decreased [30].

The number of patients who determined knee edema, pain during passive flexion/extension was significantly reduced, and local pain in the pes anserinus, lateral ligament, and knee joint was reduced. Hydrolyzate tolerance was good; not a single patient discontinued treatment due to an adverse reaction. Adverse events were noted in two patients as skin allergic reactions. Taking antihistamines allowed eliminating it and safely complete the course of treatment without any negative consequences [47]. The data obtained indicate the high efficiency of the hydrolyzate of the cartilage of hydrobionts in OA: 90% of patients at the end of the course rated the effect of the dietary supplement as good or excellent.

The inadequacy of traditional and non-traditional OA treatment practices has led cell therapy to the leading positions. The loss of articular cartilage is the main consequence of OA and it occurs in a compartment that is immunotolerant, available for cell delivery. In the 1990s, Brittberg et al. confirmed the possibility of articular cartilage restoration with cell therapy within the framework of the autologous chondrocyte implantation (ACI). ACI involves extracting chondrocytes from the weightless part of the intact articular areas, expanding them in culture, and transplanting them into focal defects in the affected joint [27]. Possibly due to the loss of chondrogenic capacity after in vitro expansion, along with the difficulty of sustained engraftment and the rapid loss of any precursors that take root in the superficial zone due to ongoing trauma, ACI therapy or matrix-induced ACI treatment (MACI) have had modest results when used in patients for the treatment of osteochondral lesions accompanying OA [28]. The use of readily available, highly proliferative, and multipotent mesenchymal stromal cells (MSC) has become the transition point of cell therapy from autologous chondrocytes. Although there have been recent studies evaluating adjuvant growth factors and new scaffold technologies, MSC infusion using direct intra-articular injections and osteochondral grafts accompanied by scaffolds or matrices is still not confirmed in clinical trials. The approach in which direct surgical injury to the articular surface stimulates the predominant fibrovascular inflammatory response by mobilizing endogenous mesenchymal precursors is called microdestruction.

The ex vivo production of MSC with a reparative ability made it possible to develop methods for direct injection of the drug locally into the joint. MSC, which includes mesenchymal stromal cells, drug signaling cells, multipotent stromal cells, can differentiate into three types of tissues (cartilage, bone, and adipose) and exhibit the ability to self-renew and multiply rapidly. MSCs have paracrine anti-inflammatory and immunomodulatory properties [29,30]; can be collected from the bone marrow (biopsy or aspirate), stromal vascular fraction of adipose tissue [16,32,48].

A total of 44 studies on intra-articular injections of MSC obtained from bone marrow, adipose tissue, and umbilical cord for the OA treatment were analyzed in a review [49]. Intra-articular injection of all three MSC cell samples has been proven to be effective and safe in the treatment of OA with minimal side effects. Pers et al. [34] evaluated 20 full-text articles (systematic reviews, comprehensive reviews, clinical reviews and meta-analyzes) published from 2006 to 2016, which provide data on the treatment of cartilage damage using MSC [34].

A group of scientists studied three doses of MSCs from adipose tissue (2 × 10^6^, 10 × 10^6^, 50 × 10^6^ of cells) [35]. It is also believed that MSCs can work through innate and adaptive immune modulation [36].

Currently, MSC procedures are quite expensive and not fully covered by most health insurance. The cost of one course of treatment for OA with stem cells in the United States is more than $5000 [37,39].

Vitamin D deficiency affects more than a billion people [39]. Especially low levels of this vitamin are observed at extreme latitudes and in the winter months, which is explained by the role of sunlight in the production of vitamin D in vivo [28]. Several proinflammatory factors, including the soluble IL-6 receptor and C-reactive protein, have their peak expression in the winter months, while the peak expression of the vitamin D receptor occurs in the summer months [38]. It was found that some diseases, including OA, have similarities in seasonality and geography. There are results of epidemiological observations that support these conclusions [31].

A certain role in the production regulation of matrix metalloproteinases and prostaglandin E2 can be played by vitamin D receptors, which are present on chondrocytes. The synthesis of proteoglycans in mature chondrocytes is stimulated by vitamin D. Bone tissue formed with a deficiency of this vitamin may be predisposed to the development of OA [50].

Despite these observations, four RCTs of vitamin D in OA have been performed in the USA [40], India [41], the United Kingdom, and Australia. None of them showed structural or symptomatic benefits in OA.

Results of a placebo-controlled study (McAlindon with colleagues) in the United States with 146 women (mean age 62.4) were described in work [4]. There were no significant improvements in knee OA symptoms, functional status, or cartilage structure associated with vitamin D supplementation.

In the aforementioned placebo-controlled study [44], despite an increase of 25-OH-vitamin D in serum (approximately 20 to 30 μg/L) in the treatment group of 474 participants over 50 years of age, there was no significant symptomatic or radiological changes in knee OA after 3 years of treatment.

A recent systematic review [45] investigated the correlation between vitamin D deficiency and knee OA. Although the results of the reviewed 11 studies established some relationship between vitamin D and OA of the knee joint, it is so ambiguous that it cannot be evidence of this association. The results included in the review [50] were mainly obtained in segmental, except for two randomized, clinical trials. There was a prominent variability in the relationship between vitamin D and knee OA.

It is known that periodontal tissue is closely related to connective tissue. It is believed that vitamin D has anti-inflammatory and antimicrobial effects, which may be a link between the known interaction of periodontitis (CP) and coronary heart disease (CHD) [51]. The amount of vitamin D received by patients, as in the case of OA, had a significant effect on the periodontal tissue, as well as on the connective tissue [52].

The trial [50] included 118 patients with knee OA who were randomly divided into treatment groups with glucosamine, MSC, the combination of these drugs, and placebo. Within 12 weeks, all groups, except for placebo, showed positive dynamics in pain and Lequesne functional index.

The effectiveness of GA and chondroitin in various combinations with MSC (MSC only with GA and/or with chondroitin and/or with placebo) was studied on a group of 147 patients with early symptomatic OA of the knee joint (Kellgren and Lawrence Classification I–II) [53]. An improvement in terms of VAS and WOMAC pain scores was established in the MSC-treated groups compared to other therapeutic prescriptions.

Cochrane review [40] studied 4 randomized, placebo-controlled, staged therapies for hip and knee OA. The review described the history of 656 patients and showed a significant improvement in mobility, but with little effect on pain (4 to 100 mm VAS) compared with placebo. No significant side effects were identified, but the quality of the data collection methodology was poor, as evidenced by the moderate degree of sample heterogeneity (I^2^ = 54%).

## 3. Conclusions

In this review, OA was classified by severity, the effectiveness of known drugs with chondroprotective properties on the course of osteoarthritis was studied. The main types of classification of OA were considered. Sources of collagen-containing industrial animal and plant materials were identified. The mechanisms of action of chondroprotective drugs (chondroitin sulfate, glucosamine sulfate or hydrochloride, hyaluronic acid, glycosaminoglycan, extraction preparations from animal or plant raw materials), their composition and properties were described. It has been established that bone and cartilage metabolism correctors based on chondroitin sulfate have chondroprotective and chondrostimulating effects. Currently, the treatment of OA is shifting towards cell therapy. Cell therapy has switched from autologous chondrocytes to the use of readily available, highly proliferative, and multipotent mesenchymal stromal cells. World studies show that there are vitamin D receptors on chondrocytes that play an important role in the regulation of the production of matrix metalloproteinases and prostaglandin E2. The severity of the problems of treatment and prevention of degenerative-dystrophic processes of connective tissue proves the relevance of ongoing research on this topic.

## Figures and Tables

**Table 1:** Kellgren and Lawrence system for classification of osteoarthritis (OA).

Grade	Degree	Features
0	None	Absence of AO signs
1	Doubtful	Possible osteophytes
2	Minimal	Definite osteophytes, intact joint
3	Moderate	Moderate narrowing of joint space
4	Severe	Severe sclerosis of the subchondral bone
